# N-terminal functional domain of Gasdermin A3 regulates mitochondrial homeostasis via mitochondrial targeting

**DOI:** 10.1186/s12929-015-0152-0

**Published:** 2015-06-24

**Authors:** Pei-Hsuan Lin, Hsien-Yi Lin, Cheng-Chin Kuo, Liang-Tung Yang

**Affiliations:** Institute of Cellular and System Medicine, National Health Research Institutes, 35 Keyan Rd., Zhunan, Miaoli County 35053 Taiwan; Graduate Institute of Molecular Systems Biomedicine, China Medical University, 91 Hsueh-Shih Rd, Taichung, 40402 Taiwan

**Keywords:** Gasdermin A3, Mitochondria, Reactive oxygen species, Cell death, Mitochondrial permeability transition

## Abstract

**Background:**

The epidermis forms a critical barrier that is maintained by orchestrated programs of proliferation, differentiation, and cell death. Gene mutations that disturb this turnover process may cause skin diseases. Human *GASDERMIN A* (*GSDMA*) is frequently silenced in gastric cancer cell lines and its overexpression has been reported to induce apoptosis. *GSDMA* has also been linked with airway hyperresponsiveness in genetic association studies. The function of GSDMA in the skin was deduced by dominant mutations in mouse *gasdermin A3* (*Gsdma3*), which caused skin inflammation and hair loss. However, the mechanism for the autosomal dominance of *Gsdma3* mutations and the mode of Gsdma3’s action remain unanswered.

**Results:**

We demonstrated a novel function of Gsdma3 in modulating mitochondrial oxidative stress. We showed that Gsdma3 is regulated by intramolecular fold-back inhibition, which is disrupted by dominant mutations in the C-terminal domain. The unmasked N-terminal domain of Gsdma3 associates with Hsp90 and is delivered to mitochondrial via mitochondrial importer receptor Tom70, where it interacts with the mitochondrial chaperone Trap1 and causes increased production of mitochondrial reactive oxygen species (ROS), dissipation of mitochondrial membrane potential, and mitochondrial permeability transition (MPT). Overexpression of the C-terminal domain of Gsdma3 as well as pharmacological interventions of mitochondrial translocation, ROS production, and MPT pore opening alleviate the cell death induced by Gsdma3 mutants.

**Conclusions:**

Our results indicate that the genetic mutations in the C-terminal domain of Gsdma3 are gain-of-function mutations which unmask the N-terminal functional domain of Gsdma3. Gsdma3 regulates mitochondrial oxidative stress through mitochondrial targeting. Since mitochondrial ROS has been shown to promote epidermal differentiation, we hypothesize that Gsdma3 regulates context-dependent response of keratinocytes to differentiation and cell death signals by impinging on mitochondria.

**Electronic supplementary material:**

The online version of this article (doi:10.1186/s12929-015-0152-0) contains supplementary material, which is available to authorized users.

## Background

The epidermis forms a physical barrier to prevent water loss and an immune barrier to defend against environmental insults. This barrier function is maintained by the epithelial keratinocytes which undergo constant proliferation, differentiation, and cell death. Gene mutations which disturb this turnover process are considered as the risking factors for skin diseases [1].

Human *GASDERMIN A* (*GSDMA*) is expressed primarily in the epithelial cells of the skin, upper gastrointestinal tract, and the lung [2–4]. The function of *GSDMA* is in dispute. *GSDMA* is frequently silenced in gastric cancer cell lines [5] and has been reported to induce apoptosis when overexpressed in gastric cancer cells [6]. *GSDMA* has also been linked with airway hyperresponsiveness; polymorphisms in *GSDMA* are associated with asthma susceptibility, and risk alleles on asthma-associated locus are linked with increased *GSDMA* gene expression [2, 7, 8]. The expression of GSDMA in the epidermis and gastric epithelium is limited to the suprabasal layer; however, GSDMA expression was also found in both basal and differentiating cells of the airway and forestomach epithelium [2, 9]. Interestingly, an expansion of GSDMA-expressing cells was found in the epidermis overlying the tumor, suggesting that *GSDMA* may mediate certain stress responses [9].

The function of *GSDMA* in the skin is unclear. Among three *GSDMA* orthologs (*Gsdma1-3*) in mice, *Gsdma1* and *Gsdma3* are expressed in the differentiating keratinocytes of the epidermis and hair follicles [10]. Only mice bearing mutations in *Gsdma3* were found to display autosomal dominant phenotypes, including epidermal hyperplasia, abnormal hair follicle differentiation, and progressive hair loss [11]. The underlying mechanism of these defects remains unexplained, as studies using different mutant mouse strains lead to different conclusions. Roles for *Gsdma3* in epidermal proliferation and differentiation [9, 12–15], apoptosis and necrosis [16, 17], and inflammation-mediated hair follicle destruction [18, 19] have been proposed.

**Fig. 1:**
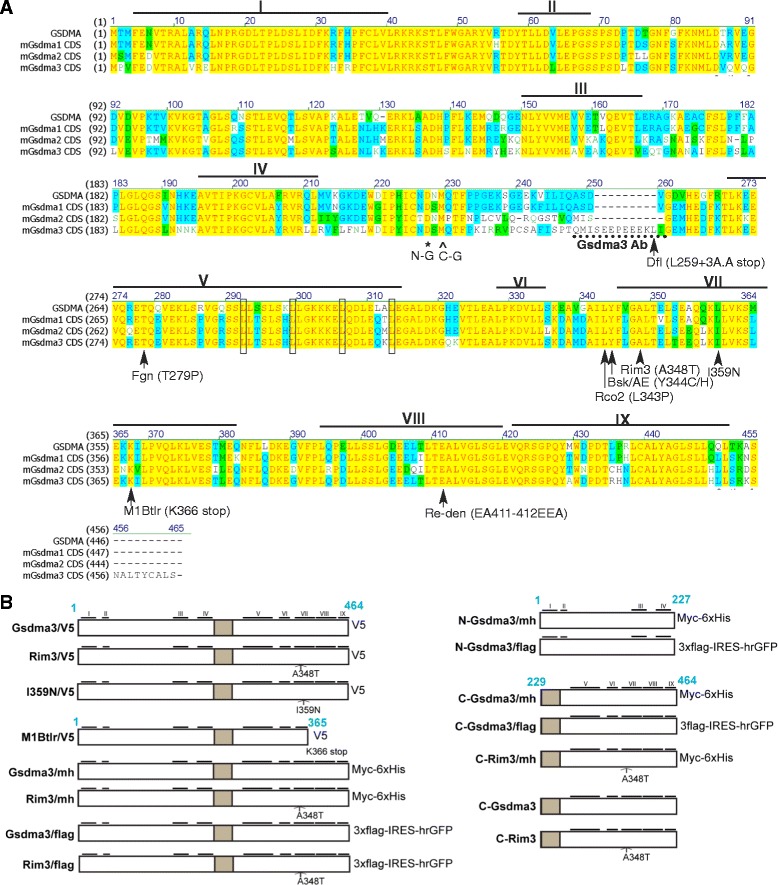
Illustration of the Gsdma3 mutants and constructs used in this study. (**a**) Multiple amino acid sequence alignment of human GSDMA and mouse Gsdma1-3 using Vector NTI software. The yellow residues are identical; the blue residues are conserved; and the green residues are similar. The rectangular boxes indicate putative leucine zipper motifs. Nine conserved leucine-rich regions across the family members are indicated with horizontal bars with Roman numerals (based on [4]). The asterisk indicates the end of the N-Gsdma3 construct, which contains the leucine-rich region I-IV; the arrowhead marks the beginning of the C-Gsdma3 construct, which contains the leucine-rich region V-XI. The mutation sites in Gsdma3 are indicated with arrows with amino acid substitutions, and the type of mutation is shown in parentheses. The dotted underlining indicates the peptide sequence used to generate the rabbit polyclonal antibody against Gsdma3*.* (**b**) A schematic representation of the Gsdma3 and mutant Gsdma3 constructs. The N-terminal (A.A.1-227, hereafter referred to as N-Gsdma3) and C-terminal (A.A. 229-464, hereafter referred to as C-Gsdma3) portions are indicated. The gray box highlights the linker region between the putative N- and C-terminal domains. The different types of C-terminal tags are labeled

The Gsdm/GSDM family proteins are characterized by the presence of a Gasdermin domain (PFAM identifier PF04598), which contains nine highly conserved leucine-rich regions distributed throughout the entire protein [4]. Three spontaneous mutations and six ENU-induced mutations have been characterized in *Gsdma3* gene (Fig. 1a). Interestingly, all the dominant mutation sites were located in the C-terminal portion of Gsdma3, indicating a critical role of this segment. Whether the dominant phenotypes of these *Gsdma3* mutants are due to haploinsufficiency or gain-of-function mutations has not been examined. The haploinsufficiency theory proposes that the mutant protein may be rapidly degraded by missense-mediated instability, whereas the gain-of-function theory proposes that these dominant mutations may generate a constitutively activated protein.

Mitochondrial membrane permeabilization is a common step in mitochondria-mediated cell death. One type is initiated at the mitochondrial outer membrane by oligomerization of the pore-forming Bak and Bax, which leads to the release of the cytochrome c and AIF from the mitochondrial intermembrane space into the cytosol toinduce caspase-dependent and -independent apoptosis. The other type is initiated at the mitochondrial inner membrane by opening of the mitochondrial permeability transition (MPT) pore, which leads to mitochondrial swelling and cell death through apoptosis or necrosis depending on the extent of the MPT. The putative MPT pore complex contains the voltage-dependent anion channel (VDAC), the adenine nucleotide translocase (ANT), and cyclophilin D (CypD) [20]. MTP pore opens in response to increased Ca^2+^ concentrations in the mitochondrial matrix. Moreover, oxidative stress, mitochondrial depolarization, and depletion of adenine nucleotide may sensitize the MPT pore to Ca^2+^ concentrations [21].

Although dominant mutations of *Gsdma3* have been associated with abnormal skin phenotypes, the mechanism for the autosomal dominance of *Gsdma3* mutations and the mode of Gsdma3’s action remain unanswered. Here, we demonstrated that the mutations of Gsdma3 disrupted the interactions between the N- and C-terminal domains. The unmasked N-terminal functional domain was then delivered to mitochondria, where it induces mitochondrial oxidative stress and cell death. Our data suggest a novel function of Gsdma3 in regulating mitochondrial homeostasis, which potentially involves in cellular differentiation and cell death.

## Methods

### Cells and reagents

HaCaT (human epidermal keratinocytes) and HEK293T (human kidney epithelial cells) were grown in DMEM (Biowest, Kansas City, MO, USA) plus 10 % FBS (Biowest) and 1X penicillin/streptomycin (Biowest). DNA transfection of HEK293T and HaCaT cells was carried out using the PolyJet and GenJet transfection reagents (SignaGen laboratories), respectively. Radicicol (Rad), N-acetyl-L-cysteine (NAC), bongkrekic acid (BkA), cyclosporin A (CsA), NecroX-5 (Nex-5), ABT-888, and E-64D were purchased from Enzo life Sciences; Necrostatin-1 (Nec1) and desipramine (Dpa) were from Santa Cruz Biotechnology.; z-VAD-fmk was from InvivoGen.

### Plasmid construction

An illustration of the *Gsdma3* mutations and the constructs used in this study is provided in Fig. 1b. For the V5-tagged constructs, the corresponding cDNAs in pENTR2B were recombined with pCDNA3.2/V5/DEST (Invitrogen) using LR clonase (Invitrogen). The Gsdma3/V5 construct was constructed through PCR amplification with primers bearing restriction sites for insertion of Gsdma3 cDNA (Genecopeia ID#Mm1921) without a stop codon and subsequent cloning into the SalI/EcoRI sites of pENTR2B (Invitrogen). The *Rim3*-type (*Gsdma3* A384T) and *I359N*-type constructs were generated via site-directed mutagenesis (1124 G to A for Rim3-type and 1158 T to A for I359N-type) of *Gsdma3* cDNA in pENTR2B, following the instructions for the QuikChange Site-Directed Mutagenesis kit (Agilent Technologies). The *M1Btlr*-type (Gsdma3 K366 stop) construct was constructed via PCR amplification of a.a. 1-365 of the Gsdma3 cDNA and cloned in pENTR2B. For Gsdma3/mh and Rim3/mh constructs, the corresponding SalI-NotI cDNA fragments from the pENTR2B constructs were cloned into XhoI/NotI sites of pCDNA3.1-myc-His(-)B (Invitrogen). For the rest of myc-tagged constructs, the corresponding cDNA sequences were PCR amplified and cloned into pCDNA3.1-myc-His(-)A to generate a myc/6xHis tag. For the flag-tagged-ires-hrGFP constructs, the corresponding cDNA sequences were PCR amplified and cloned into pIRES-hrGFP-1a (Agilent Technologies) to obtain a 3xflag tag and achieve IRES-mediated GFP expression. Untagged C-Gsdma3 and C-Rim3 were constructed through PCR amplification (with the stop codon sequence) and cloned into pCDNA3.1-myc-His(-)A. All constructs were verified through DNA sequencing and western blot analysis. The primer sequences for PCR cloning and mutagenesis are listed in Table 1.

**Table 1:** Primer sequences for cloning and mutagenesis of the constructs

Construct name	Method	Primer sequences (5’ → 3’)
Gsdma3	PCR cloning	forward, AGCGTCGACAGACAATGCCTGTGTTTGAGG;
-pENTR2B		reverse, CCGGAATTCGAAGATAGAGCACAATAAGTAAGTGC
Gsdma3I359N -pENTR2B	mutagenesis	forward, GCTAACTGAAGAACAACTGAAGAATCTAGTAA AATCCTTGGAGA
		reverse, TCTCCAAGGATTTTACTAGATTCTTCAGTTGTTCTTCAGTTAGC
Gsdma3^M1Btlr^	mutagenesis	forward, GCTAACTGAAGAACAACTGAAGAATCTAGTAA AATCCTTGGAGA;
-pENTR2B		
		reverse, TCTCCAAGGATTTTACTAGA TTCTTCAGTTGTTCTTCAGTTAGC
Gsdma3^M1Btlr^	PCR cloning	forward, AGCGTCGACAGACAATGCCTGTGTTTGAGG;
-pENTR2B		
		reverse, GCGGA ATTCCCCTCCAAGGATTTTACTAG
N-Gsdma3	PCR cloning	forward, AGCGAATTCAGACAATGCCTGTGTTTGAGG;
-pCDNA3.1-myc-His(-)A		reverse, AGCGAATTCCGCTGTCATTGCAAATGTACGG
C-Gsdma3	PCR cloning	forward, AGCGAATTCATATGCAAACCTTCCCTAAGATCAGG;
-pCDNA3.1-myc-His(-)A		reverse, CCGGAATTCCAAGATAGAGCACATAAGTAAGTGC
C-Gsdma3^Rim3^	PCR cloning	forward, AGCGAATTCATATGCAAACCTTCCCTAAGATCAGG;
-pCDNA3.1-myc-His(-)A		reverse, CCGGAATTCCAAGATAGAGCACATAAGTAAGTGC
Gsdma3	PCR cloning	forward, TCCGCGGCCGCACCATGCCTGTGTTTGAGGAT;
-pIRES-hrGFP-1a		
		reverse, CCGGAATTCCAGATAGAGCACAATAAGTAA
Gsdma3Rim3	PCR cloning	forward, TCCGCGGCCGCACCATGCCTGTGTTTGAGGAT;
-pIRES-hrGFP-1a		
		reverse, CCGGAATTCCAGATAGAGCACAATAAGTAA
N-Gsdma3	PCR cloning	forward, TCCGCGGCCGCACCATGCCTGTGTTTGAGGAT;
-pIRES-hrGFP-1a		
		reverse, CCGGAATTCCGCTGTCATTGCAAATGTACGG
C-Gsdma3	PCR cloning	forward, TCCGCGGCCGCACGATGCAAACCTTCCCTAAG;
-pIRES-hrGFP-1a		
		reverse, CCGGAATTCCAGATAGAGCACAATAAGTAA
C-Gsdma3 (no tag)	PCR cloning	forward, AGCGAATTCATATGCAAACCTTCCCTAAGATCAGG;
-pCDNA3.1-myc-His(-)A		reverse, CCGGAATTCAAAGATAGAGCACATAAGTAAGTGC
C-Gsdma3^Rim3^ (notag)	PCR cloning	forward, AGCGAATTCATATGCAAACCTTCCCTAAGATCAGG;
-pCDNA3.1-myc-His(-)A		reverse, CCGGAATTCAAAGATAGAGCACATAAGTAAGTGC

### Flow cytometry analysis

HEK293T cells were seeded at a density of 2x10^5^ cells/well in 6-well plates and were transfected with pIRES-GFP-1a vector-based constructs as indicated in the figures. The transfection efficiency was usually 50-60 % based on the GFP signal from pIRES-hrGFP-1a-transfected cells. Sixteen to twenty hours after transfection, the cells were triturated off the plate in HBSS. For mitochondrial ROS detection, the cells were loaded with 2.5 μM MitoSox Red (Molecular Probe) in HBSS, incubated in a 5 % CO_2_ incubator at 37 °C for 15 min, washed with PBS and then analyzed on a FACSCalibur (BD Biosciences) using CellQuest Pro software. To assess the mitochondrial membrane potential and plasma membrane integrity, cells were washed with HBSS, loaded with 1 nM DiIC_1_(5) (Molecular Probe) in HBSS, and incubated in a 5 % CO_2_ incubator at 37 °C for 15 min. The cells were then washed with PBS, and 7-aminoactinomycin D (7-AAD, 200 ng/ml, Molecular Probe) was added 10 min before flow cytometry analysis. For quantitative analysis, an equal number (approximately 20,000 cells) of GFP^+^ cells was collected for data analysis using FlowJo software (Tree Star, Inc.). Transfected cells were treated with pharmacological inhibitors 16 hours before flow cytometry.

### Western blot analysis, immunoprecipitation, subcellular fractionation, and proteinase K treatment

HEK293T cells in 6 cm dish were transfected with different combinations of plasmid constructs, as indicated in the figures; the total amount of plasmid in each transfection was kept constant across the samples through co-transfection with the pCDNA3 vector plasmid. Western blotting and immunoprecipitation were carried out as previously described [22]. The mitochondria/cytosol fractionation of transfected cells was carried out using a Mitochondria Isolation Kit for cultured cells (Pierce) according to the manufacturer’s protocol. The measurement of cytochrome c released from the mitochondria was performed according to the protocol described previously [23]. Proteinase K treatment of fractionated mitochondria was performed in SHE buffer (0.25 M sucrose, 1 mM EDTA, 20 mM HEPES-NaOH, pH 7.4) in the presence or absence of 5 μg/ml proteinase k (PK) for 30 min on ice. The sources and dilutions of the primary antibodies used in these assays are listed in Table 2. An anti-Gsdma3 antibody was raised in rabbits using the peptide sequence QMISEEPEEEKLI (Yao-Hong Biotechnology, New Taipei City, Taiwan).

**Table 2:** List of the primary antibodies and dilutions used for western blotting/ immunoprecipitation (WB/IP) and immunostaining (ICC)

Antigen	Antibody	Species	Company	WB/IP	ICC
V5	monoclonal	mouse sv5-pk	Santa Cruz	1:500/IP(2 μg)	1:300
V5	polyclonal	rabbit	Genetex	1:5000	1:200
myc	monoclonal	mouse 9E10	Santa Cruz	1:1000/IP(2 μg)	1:200
flag	monoclonal	mouse M2	Agilent Tech	1:2000/IP(2 μg)	
Cox4	polyclonal	rabbit	Genetex	1:1000	
Tom20	polyclonal	rabbit	Santa Cruz		1:500
Gsdma3	polyclonal	rabbit	Home made	1:500	1:200
β-actin	polyclonal	rabbit	Genetex	1:5000	
GAPDH	Polyclonal	rabbit	Genetex	1:5000	
Hsp90α/β	polyclonal	rabbit	Santa Cruz	IP (2 ug)	
Hsp90β	polyclonal	rabbit	Genetex	1:2000	
Hsp70 1A	polyclonal	polyclonal rabbit	Genetex	1:2000	
STIP1/Hop	polyclonal	rabbit	Genetex	1:2000	
TRAP1	monoclonal	mouse	Genetex	IP (2 ug)	
TRAP1	polyclonal	rabbit	Genetex	1:4000	
AKT1	polyclonal	rabbit	Millipore	1:2500	
Tom70	monoclonal	mouse	Santa Cruz	1:2000/IP(2 ug)	

### Immunostaining, apoptosis/necrosis detection, and TUNEL assays

Immunostaining was performed following the standard procedures as described elsewhere [24]. The sources and dilutions of the primary antibodies used in these assays are listed in Table 2. The fluorescence signals were imaged using an Olympus DP71 CCD device attached to an Olympus BX51 microscope with a DP controller and DP manager software or with a Leica TCS SP5 II confocal microscope system with Leica LAS AF software. A GFP-Certified Apoptosis/Necrosis detection kit (Enzo) was used to analyze apoptosis/necrosis according to the manufacturer’s instructions. TUNEL assays were conducted using the DeadEnd Fluorometric TUNEL system (Promega) following the manufacturer’s instructions.

### Proteomics analysis

The protein bands detected via SDS-PAGE by silver staining using the SiverQuest staining kit (Invitrogen) were manually excised from the gel, reduced, alkylated and trypsin digested as described previously [25]. A nanoAcquity UPLC-nano-ESI-MS/MS analysis was applied to the extracted peptides using a Synapt G2 HDMS mass spectrometer (Waters Corp.). Peptides were trapped and desalted in a nanoAcquity UPLC®Trap Column (180 μm × 20 mm, 5 μm Symmetry® C18) using 99 % solvent A/1 % solvent B at 5 μL/min for 3 min and were then separated in a nanoACQUITY UPLC BEH 130 C18 reverse-phase column (Waters Corporation) using a linear-gradient of 3-60 % solvent B at 200–300 nl/min over 85 min. Data-directed analysis was used for data acquisition. A 1-second scan was applied to obtain an MS survey at a range of 300-2000 m/z. The 5 most abundant ions with a charge state of 2^+^, 3^+^, or 4^+^ were selected for MS/MS analysis and were dynamically excluded for 70 seconds. MS/MS spectra (range 50-2000 m/z, 1.2 sec/scan) were obtained by ramping the collision cell energy from 15-30 V for low molecular weights and 30-55 V for high molecular weights. The mass accuracy was 20 ppm for the parent ion and 0.25 Da for the fragment ions. The acquired raw data were submitted to the ProteinLynx Global Server and exported as PKL files for human protein database searches at UniProt using a Mascot Daemon 2.4.1 server. The data were filtered using a MASCOT P < 0.05.

### Western blot quantification, confocal microscopy processing, and statistical analysis

Western blot X-ray films were scanned using an ArtixScan 2500f scanner (Microtek), and the digital image files were analyzed using ImageJ (NIH) according to the Gel Analysis method documented online. Images acquired in a confocal microscopy system with a z-step of approximately 1 μm were processed to determine the degree of colocalization using the Imaris software (Bitplane). Briefly, the degree of immunofluorescence overlap between the target protein and the mitochondrial marker, represented by % region of interest (ROI) colocalized with Tom20, was calculated from a minimum of ten cells. The ROI was determined by masking the images such that the pixels selected in each channel closely outlined the area of immunofluorescence staining; the threshold was then calculated by the program using the default settings. A colocalization channel was created, and the fraction of the ROI that colocalized with the mitochondrial marker was determined for each image. Statistical analysis was carried out in Excel 2003 (Microsoft), and statistical significance was determined with two-tailed Student’s *t* tests for comparing two independent groups, or with one-way ANOVA using α = 0.05 for comparing more than two groups. In all experiments P < 0.05 is considered statistically significant; *, P < 0.05; **, P < 0.01; ***, P < 0.001.

## Results

### The N-terminal domain of Gsdma3 harbors the cell death-inducing ability

Sequence alignment of human and mouse GSDMA/Gsdma family members suggests that Gsdma3 consists of highly conserved N-terminal and C-terminal domains that are connected by a variable linker region. Notably, known autosomal dominant mutations, including *Bsk*, *Dfl, Re-den*, *Fgn*, *Rco2*, *Rim3*, *AE*, *M1Btlr*, and *I359N*, are located in the C-terminal domain (Fig. 1a). To investigate the structure-function relationship of Gsdma3, we generated various expression constructs to express wild-type, truncated-type, and mutant-type variants with different tagging sequences (Fig. 1b). Since Rim3-type (A348T) mutation changes the highly conserved residue in the seventh leucine-rich region (LRR) of Gsdma3 protein and six out of nine dominant mutations are located in this LRR, we used mainly this mutant protein (Gsdma3^Rim3^) as a paradigm for functional study of the missense mutation.

**Fig. 2:**
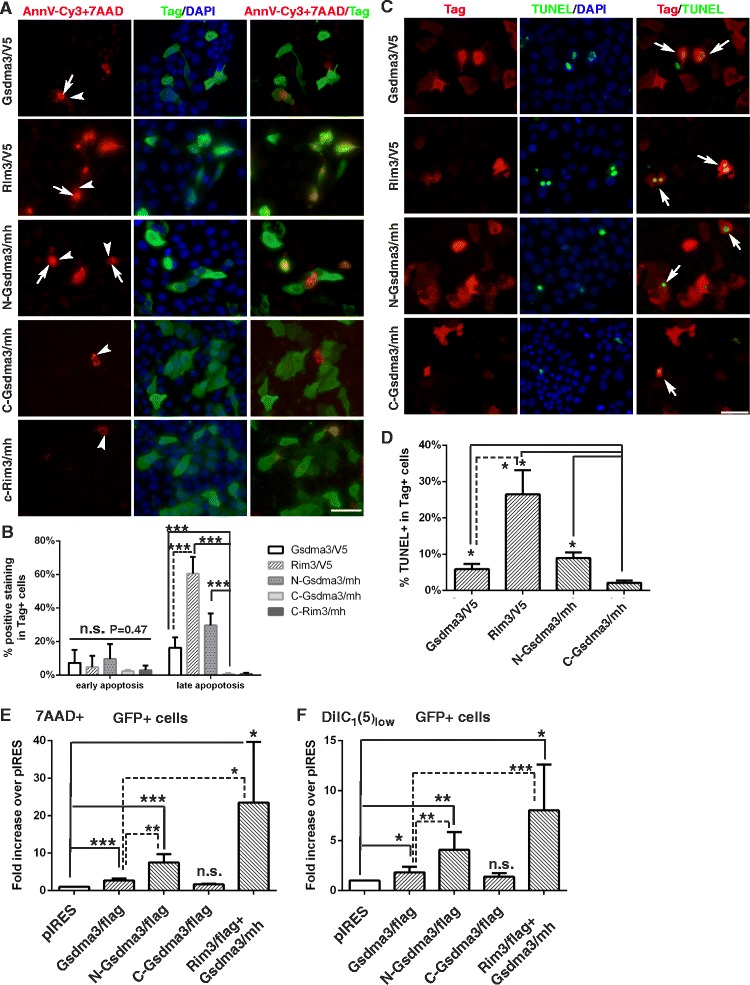
The N-terminal domain of Gsdma3 harbors the cell death-inducing ability of Gsdma3. (**a**) Immunostaining of HaCaT cells transfected with the indicated plasmid constructs. Cells were live-cell stained with Annexin V-Cy3 and 7-AAD and immunostained for the tagged constructs. Arrows, Annexin V-Cy3 cell surface staining (red); arrowheads, 7-AAD nuclear staining (red); Tag-specific staining (green). (**b**) Quantification of the apoptosis/necrosis staining. The percentage of Annexin V^+^ (early apoptosis) cells and the percentage of Annexin V^+^7AAD^+^ (late apoptosis) cells among the tag^+^ cells (mean + s.d., n > 5 microscopy fields from three independent experiments) are shown for each transfected construct. Vector-transfected cells exhibited 0.1 ± 0.2 % early apoptosis and 1.4 ± 0.9 % late apoptosis. (**c**) TUNEL staining in HaCaT cells transfected with the indicated plasmid constructs, followed by immunostaining for the tagged constructs. TUNEL (green) and tag-specific (red) staining results are shown. DAPI counterstains the nuclei in blue. Scale bar, 50 μM. (**d**) Quantification of the TUNEL stainings by the percentage of TUNEL^+^ cells among tag^+^ cells (mean + s.d., n > 6 microscopy fields from two independent experiments). Vector-transfected cells displayed a 1.32 ± 0.57 % TUNEL^+^ signal. (**e**, **f**) Flow cytometry analysis of cell death and mitochondrial membrane potential (MMP) depolarization. HEK293T cells were transfected with flag tagged-constructs and the vector pIRES-hrGFP-1a, stained with 7-AAD and DiIC_1_(5), followed by flow cytometry analysis. Quantifications results show the fold increase (mean + s.d., n >3) in the fraction of dead (7AAD^+^) GFP+ cells and mitochondrial membrane potential depolarization (DiIC_1_(5)_low_) GFP^+^ cells over that of pIRES-GFP-1a vector-transfected cells (Dead, 1.88 ± 0.7 %, Low MMP, 4.76 ± 2.14 %).*, P < 0.05; **, P < 0.01; ***, P < 0.001; n.s., not significant

Previous overexpression study of GSDMA and Gsdma3 implicated a role in apoptosis [6, 16]; we therefore expressed wild-type and mutant Gsdma3 in epithelial cells and examined the cell death phenotypes. HaCaT cells transfected with expression vectors for wild-type and mutant Gsdma3 were first live-cell stained with Annexin V-Cy3 plus 7-AAD, and then immunostained for the tagged protein (Fig. 2a). We found that the cells expressing wild-type Gsdma3 (Gsdma3^wt^), N-terminal portion of Gsdma3 (N-Gsdma3) or Gsdma3^Rim3^ displayed slightly higher levels of early apoptosis staining (Annexin V^+^) than that of the cells expressing the C-terminal portion of Gsdma3 (C-Gsdma3) and C-terminal portion of Gsdma3^Rim3^ (C-Gsdma3^Rim3^), but it is not statistically significant (P = 0.47). The expression of C-Gsdma3 and C-Gsdma3^Rim3^ induced very little late apoptosis (Annexin V^+^ 7AAD^+^), and Gsdma3-expressing cells (16.4 ± 6.1 %), N-Gsdma3-expressing cells (29.6 ± 7.2 %), and Gsdma3^Rim3^-expressing cells (60.5 ± 9.9 %) displayed higher level of late apoptosis staining than C-Gsdma3-expressing cells (P < 0.005) (Fig. 2b). The TUNEL assays were also performed in transfected HaCaT cells to detect the late apoptosis (Fig. 2c). Quantification of the TUNEL staining results revealed that Gsdma3-, N-Gsdma3- and Gsdma3^Rim3^-expressing cells displayed a higher level of TUNEL^+^ staining than that of C-Gsdma3-expressing (P < 0.5) (Fig. 2d). Interestingly, we found that Gsdma3^Rim3^-expressing cells had a higher late apoptosis staining than Gsdma3-expressing cells (Fig. 2b, d).

To quantify the cell death status in a more objective manner, we used flow cytometry to analyze the plasma membrane integrity (7AAD permeability) and mitochondrial membrane potential (△ΨM) in HEK293T cells expressing Gsdma3 and its variants (Fig. 2e, f). The pIRES-hrGFP-1a vector was used to express flag-tagged Gsdma3 and variants together with internal ribosome entry site-driven GFP. Twenty thousand GFP+ cells for each transfection were collected for flow cytometry analysis, and we found that N-Gsdma3-expressing cells displayed a significant increase in the fractions of cell death (7AAD^+^ staining) and △ΨM depolarization than Gsdma3-expressing cells or vector-transfected cells. In contrast, the fractions of cell death and cells with △ΨM depolarization in C-Gsdma3-expressing cells were comparable to vector-transfected cells. As the GFP signal in cells transfected with flag tagged-Gsdma3^Rim3^ was below the detection limit of flow cytometry, we transfected HEK293T cells with mixtures of the flag tagged-Gsdma3^Rim3^ and the V5 tagged-Gsdma3^wt^ plasmids (1:2 ratio). Interestingly, coexpression of Gsdma3^Rim3^ and Gsdma3^wt^ resulted in greater fractions of cell death and △ΨM depolarization than did expression of Gsdma3^wt^ alone. Collectively, these results indicate that C-Gsdma3 and C-Gsdma3^Rim3^ do not cause cell death, and that N-terminal domain of Gsdma3 harbors the cell death-inducing ability and the Rim3-type (A348T) mutation in the C-terminal domain may play a regulatory role.

### Coimmunoprecipitation analyses reveal an association between the N- and C-terminal domains of Gsdma3

**Fig. 3:**
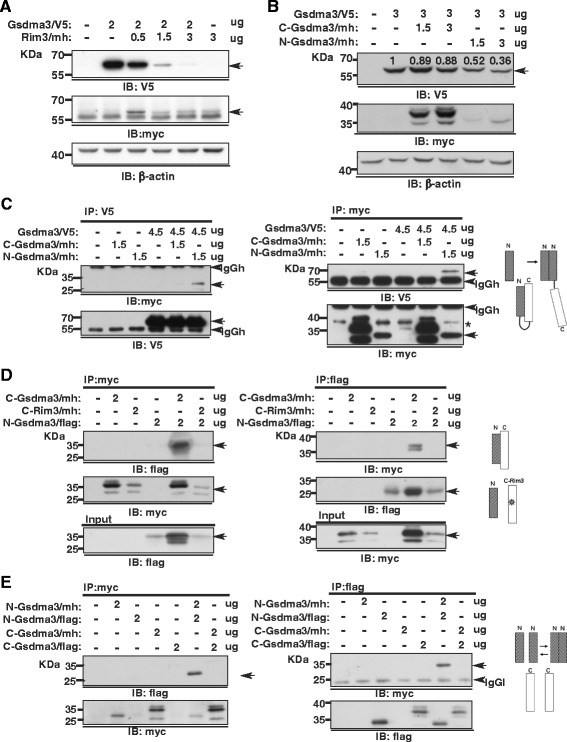
Coimmunoprecipitation analyses of the association between the N- and C-terminal domains of Gsdma3. (**a**, **b**) Immunoblot analysis of HEK293T cells transfected with the indicated plasmid constructs. Relative densitometry levels normalized to β-actin expressions are indicated. (**c**, **d**, **e**) Co-immunoprecipitation and immunoblot analysis of HEK293T cells transfected with the indicated plasmid constructs. Whole cell lysates (WCL) were split in half, immunoprecipitated and immunoblotted with the indicated antibodies. Asterisks indicate non-specific reactive bands. The molecular weight of the protein standards in kilodaltons (kDa) is indicated. Non-specific bands corresponding to the IgG heavy chain (IgGh) and light chain (IgGl) are indicated. Models of the putative domain interaction are listed in each panel

HEK293T cells transfected with V5-tagged Gsdma3 plasmid displayed an appreciable level of expression, but the cells transfected with an equal amount of V5-tagged Gsdma3^Rim3^ plasmid exhibited reduced levels of expression, possibly due to its cytotoxicity (Additional file 1: Figure S1A). Interestingly, the expression level of V5 taggd-Gsdma3 was significantly reduced as the amount of the co-transfected Rim3/mh or N-Gsdma3/mh plasmid construct increased (Fig. 3a, b). These dominant effects prompted us to investigate the interaction between Gsdma3 and its variants, which was further demonstrated by reciprocal co-immunoprecipitation analyses, where Gsdma3 was found to self-associate and associate with Gsdma3^Rim3^, but the level of Gsdma3-Gsdma3^Rim3^ complex formation was lower than that of Gsdma3^wt^ self-association (Additional file 1: Figures S1B-S1D).

Moreover, the co-immunoprecipitation results first demonstrated that the N-Gsdma3, but not C-Gsdma3, associated with full-length Gsdma3^wt^ (Fig. 3c), and that N-Gsdma3 interacted with C-Gsdma3 (Fig. 3d) and self-associated (Fig. 3e). Importantly, the Rim3-type mutation rendered C-Gsdma3 (C-Gsdma3^Rim3^) unable to associate with N-Gsdma3 (Fig. 3d). Analysis of HEK293T cells co-expressing myc-His tagged-N-Gsdma3 with untagged C-Gsdma3 or C-Gsdma3^Rim3^ confirmed that the expression of C-Gsdma3, but not C-Gsdma3^Rim3^, increased the N-Gsdma3 protein expression (Additional file 1: Figure S1E). These results suggest that the Gsdma3 protein can be stabilized by an association between its N- and C-terminal portions, which also mediates self- and intermolecular associations.

### Mitochondrial targeting of N-Gsdma3 is masked by C-Gsdma3

**Fig. 4:**
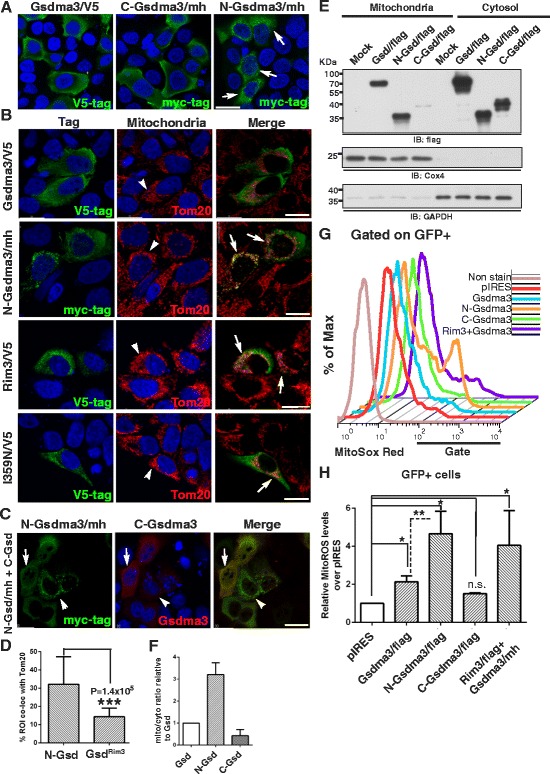
N-Gsdma3 partially colocalizes with mitochondria and induces mitochondrial ROS production. (**a**) Immunostaining of HaCaT cells transfected with the Gsdma3/V5, C-Gsdma3/mh, or N-Gsdma3/mh plasmid. Scale bar: 30 μM. (**b**) HaCaT cells transfected with Gsdma3/V5, N-Gsdma3/mh, Rim3/mh, and I359N/V5 plasmids were double immunostained for the tag and the mitochondrial marker Tom20. The region of interest (ROI) is determined by masking the images using the Imaris software such that the pixels selected in green and red channels closely outline the area of immunofluorescence staining. Arrows indicate partial colocalization; arrowheads point to mitochondria; Scale bar: 20 μM. (**c**) HaCaT cells were transfected with both the C-Gsdma3 and N-Gsdma3/mh plasmids and double immunostained for the myc tag and Gsdma3. The arrowheads indicate the cells expressing a low amount of C-Gsdma3; the arrows indicate the cells expressing a high amount of C-Gsdma3. Scale bar, 20 μM. The color coding indicates the secondary antibody labeling. DAPI counterstains the nuclei in blue. (**d**) Quantification of the colocalization of N-Gsdma3 and Gsdma3^Rim3^ with the mitochondrial marker. The bar graph shows the % ROI colocalized with Tom20 (mean + s.d., n > 10 from 5-7 z-stacked confocal images). ***, P < 0.01 (**e**) Immunoblot analysis of mitochondrial and cytosolic fractions prepared from transfected HEK293T cells. Cox4 (a mitochondrial marker) and GAPDH (a cytosol marker) immunoblots confirmed the purity of the fractionations. (**f**) Quantification of the mitochondria/cytosol distribution based on relative densitometry levels from mitochondrial and cytosolic fractions. The bar graph represents mitochondrial/cytoplasmic ratio relative to Gsd (mean + s.d., n = 3). (**g**) Flow cytometry analysis of the MitoSox Red staining in HEK293T cells transfected with the Gsdma3/flag (Gsdma3), N-Gsdma3/flag (N-Gsdma3), C-Gsdma3/flag (C-Gsdma3), Rim3/flag plus Gsdma3/mh in a ratio of 1:2 (Rim3 + Gsd), and control pIRES-hrGFP-1a (pIRES) plasmids. (**h**) Quantification of the MitoSox Red staining results by the fold increase of MitoSox Red staining in the fraction of GFP^+^ cells (mean + s.d., n = 3) over that of vector-transfected cells (average = 3.79 ± 1.53 %).*, P < 0.05; **, P < 0.01; n.s., not significant

To gain further insight into the molecular function of Gsdma3, we next investigated the subcellular localization of N-Gsdma3, C-Gsdma3, and full-length Gsdma3^wt^ in HaCaT cells. Immunostaining results from transfected HaCaT cells revealed that both Gsdma3 and C-Gsdma3 displayed diffuse cytosolic staining, while N-Gsdma3 mainly displayed a perinuclear punctate staining (Fig. 4a). Double-immunostaning of HaCaT cells transfected with Gsdma3, N-Gsdma3, Gsdma3^Rim3^, and Gsdma3^I359N^ expression plasmids (Fig. 1b) with tag-specific antibody and markers of individual organelles (ER, mitochondria, Golgi, peroxisomes, and lysosomes) showed that N-Gsdma3 as well as Gsdma3^Rim3^, and Gsdma3^I359N^ partially colocalized with the mitochondrial marker Tom20 (Fig. 4b, d). Interestingly, N-Gsdma3, Gsdma3^Rim3^, and Gsdma3^I359N^ often colocalized with fragmented mitochondria. Gsdma3^Rim3^ partially colocalized with Tom20, albeit less than N-Gsdma3 did. Remarkably, co-expressing C-Gsdma3 relocated N-Gsdma3 to the cytosol (Fig. 4c). Using immunostaining, we confirmed that N-Gsdma3 also partially colocalized with mitochondria in HEK293T and A549 lung epithelial cells (Additional file 2: Figure S2), suggesting that mitochondrial targeting of N-Gsdma3 is not a cell type-specific mechanism. To confirm mitochondrial targeting of N-Gsdma3, we performed mitochondria/cytosol fractionations of transfected HEK293T cells and immunoblotting analyses (Fig. 4e). Quantitative analysis showed that N-Gsdma3, but not C-Gsdma3, was enriched in the mitochondrial fraction (Fig. 4f). Collectively, our data suggest that the N-terminal domain of Gsdma3 targets mitochondria, while the C-terminal domain of Gsdma3 masks its mitochondrial targeting.

### N-Gsdma3 increases reactive oxygen species production in mitochondria

The fragmented mitochondria observed in the N-Gsdma3-expressing cells prompted us to use flow cytometry to examine the levels of mitochondrial reactive oxygen species (ROS) in transfected cells (Fig. 4g). We detected an increase of mitochondrial ROS level in N-Gsdma3- and Gsdma3^wt^-expressing cells compared with pIRES-hrGFP-1a vector -transfected cells (P < 0.5) (Fig. 4h). In contrast, levels of mitochondrial ROS in C-Gsdma3-expressing cells and vector control-transfected cells were comparable. Furthermore, coexpression of Gsdma3^Rim3^ and Gsdma3^wt^ (1:2 ratio) also resulted in an increase of mitochondrial ROS level over vector-transfected cells. These data indicate that unmasked N-Gsdma3 increases mitochondrial ROS production.

### C-Gsdma3 alleviates the cytotoxicity of Gsdma3 mutants

**Fig. 5:**
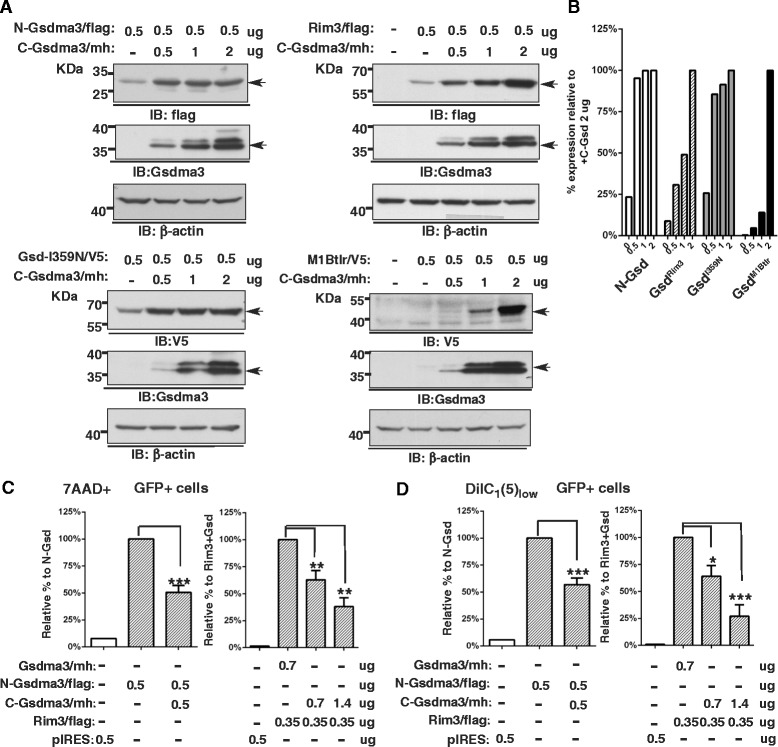
C-Gsdma3 blocks the cytotoxicity induced by Gsdma3 mutants. (**a**) Immunoblot analysis of HEK293T cells transfected with the N-Gsdma3/flag, Rim3/flag, M1Btlr/V5, or Gsdma3-I359N/V5 plasmid together with an increasing amount of the C-Gsdma3/mh plasmid. The anti-Gsdma3 antibody is more sensitive than the anti-myc-tag antibody. β-actin was used as a loading control. The asterisk indicates a non-specific band due to the long exposure. (**b**) Quantification of the immunoblots from (**a**). The bar graph shows the normalized densitometry levels relative to the sample co-transfected with 2 μg of the C-Gsdma3/mh plasmid. (**c**, **d**) Flow cytometry analysis of the 7AAD and DiIC_1_(5) staining in HEK293T cells transfected with different combinations of plasmid constructs as indicated. The bar graphs represent fractions of dead (7AAD+) cells and mitochondrial membrane potential depolarization (DiIC_1_(5)_low_) cells (mean + s.d., n = 3 in duplicates) relative to those in N-Gsdma3- or Rim3 + Gsdma3-trasnfected cells**.** pIRES vector-transfected cells were used as the staining baseline.. *, P < 0.05; **, P < 0.01; ***, P < 0.001

We reasoned that if the mutations in C-Gsdma3 disrupted the association between N-Gsdma3 and C-Gsdma3 and exposed N-Gsdma3, then exogenous expression of the C-Gsdma3 might inhibit the cytotoxicity of Gsdma3 mutants. 293 T cells were transfected with N-Gsdma3, Gsdma3^Rim3^, Gsdma3^I359N^, or Gsdma3 ^M1Btlr^ (nonsense mutation) expression plasmids together with increasing amount of C-Gsdma3 expression plasmid. We found that the expression levels of the Gsdma3 mutants were generally lower than those of wild-type Gsdma3 and that increasing C-Gsdma3 expression enhanced the protein levels of all Gsdma3 mutants tested, possibly by alleviating the toxicity of the mutant proteins (Fig. 5a). Notably, quantitative results showed that a greater amount of the C-Gsdma3 plasmid was needed to restore the maximum protein expression of Gsdma3^Rim3^ and Gsdma3^M1Btlr^ mutants (Fig. 5b). We further examined the effect of C-Gsdma3 expression on the cytotoxicity induced by N-Gsdma3 and Gsdma3^Rim3^ through flow cytometry analysis (Fig 5c, d). We found that expressing C-Gsdma3 alleviated cell death (7AAD+) and △ΨM depolarization (low DiIC_1_[5]) induced by N-Gsdma3 and Gsdma3^Rim3^ plus Gsdma3^wt^. Also, a greater amount of the C-Gsdma3 plasmid was needed to block the cytotoxicity of Gsdma3^Rim3^ plus Gsdma3^wt^. Together our results indicated that expressing C-Gsdma3 masks the cytotoxicity exerted by N-Gsdma3 or Gsdma3 mutants.

### N-Gsdma3 associates with Hsp90 and is delivered to mitochondria

**Fig. 6:**
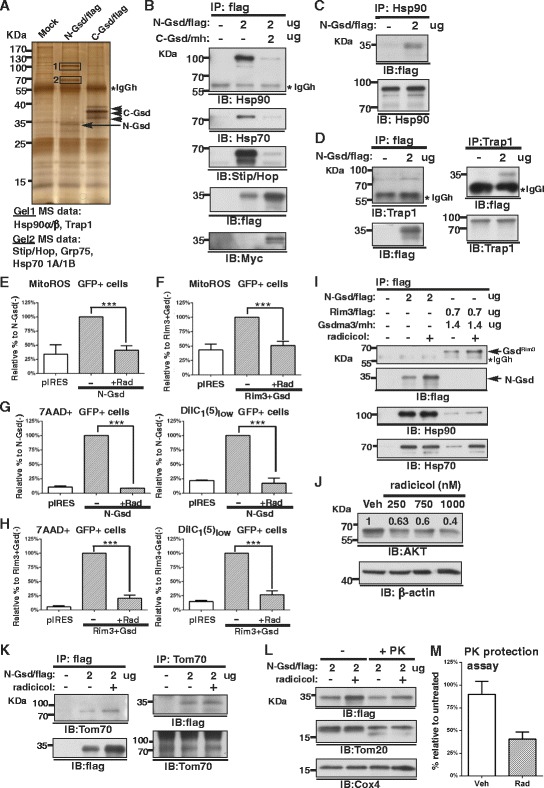
N-Gsdma3 associates with Hsp90 and the mitochondrial chaperone Trap1. (**a**) Proteomics analysis. Anti-flag immunoprecipitation followed by SDS-PAGE and silver staining of transfected HEK293T cells. Unique gel bands (Gel1 and Gel2) revealed in the N-Gsdma3 immunoprecipitate were excised out and processed for proteomics analysis. (**b**, **c**) Reciprocal co-immunoprecipitation of Hsp90 and N-Gsdma3. (**d**) Reciprocal co-immunoprecipitation of N-Gsdma3 and Trap1. (**e**, **f**) Quantification results for MitoSox Red staining. The bar graphs depict the mitochondrial ROS levels (mean + s.d., n = 3 in duplicates) in radicicol (750 nM)-treated transfected cells relative to those in vehicle-treated transfected cells. (**g**, **h**) Quantification results for 7AAD and DiIC_1_(5) staining. The bar graphs represent fractions of dead (7AAD+) cells and mitochondrial membrane potential depolarization (DiIC_1_(5)_low_) cells (mean + s.d., n = 3 in duplicates) in radicicol (750 nM)-treated transfected cells relative to those in vehicle-treated transfected cells. pIRES vector-transfected cells were used as the staining baseline. ***, P < 0.001. No apparent cell death was induced by 750 nM radicicol treatment of HEK293T cells for 20 hours. (**i**) Co-immunoprecipitation of N-Gsdma3 and Gsdma3^Rim3^ with Hsp90/Hsp70 in transfected HEK293T cells in the absence or presence of 750 nM radicicol. (**j**) Immunoblot analysis of AKT protein in HEK293T cells after treatment with radicicol (250, 750, 1000 nM) for 20 hours. β-actin was used as a loading control. Relative densitometry intensities are indicated above the protein bands. (**k**) Reciprocal co-immunoprecipitation of Tom70 and N-Gsdma3 in the absence or presence of 750 nM radicicol (**l**) Immunoblot analysis of fractionated mitochondria from transfected HEK293T cells in the absence or presence of 750 nM radicicol either left untreated (-) or treated with 5 μg/ml proteinase K (+PK). Tom20 was used as an indicator of PK treatment; Cox4 was used as the loading control. (**m**) Quantification of the PK protection assay. The bar graph depicts the densitometry levels of N-Gsdma3 in the radicicol-treated preparation relative to the untreated preparation (mean + s.d., n = 3)

To investigate how N-Gsdma3 is targeted to mitochondria and induces mitochondrial ROS production, we used immunoprecipitation and LC-mass spectrometry analysis to identify its interacting proteins (Fig. 6a, Additional file 3: Figure S3, Additional file 4: Table S1). We identified Hsp90β, Hsp90α, and Trap1 (Gel1, approximately 100 kDa) as well as Hsp70 1A, Hsp70 1B, Stip1/Hop, and Grp75 (Gel2, approximately 70 kDa) as potential N-Gsdma3-interacting proteins. By coimmunoprecipitation experiments, we demonstrated that N-Gsdma3 associated with endogenous Hsp90/Hsp70/Hop complex, while the co-expressing C-Gsdma3 disrupted the association between the Hsp90 complex and N-Gsdma3 (Fig. 6b). Reciprocal co-immunoprecipitation further confirmed the association of Hsp90 with N-Gsdma3 (Fig. 6b). In addition, the co-immunoprecipitation experiments confirmed the association between N-Gsdma3 and the mitochondrial chaperone Trap1/mtHsp75 (Fig. 6d).

Next, we used the Hsp90 inhibitor radicicol to address the role of Hsp90 association with N-Gsdma3. We found that radicicol treatment reduced mitochondrial ROS production, cell death, and △ΨM dissipation induced by N-Gsdma3 and Gsdma3^Rim3^ plus Gsdma3^wt^ (Fig 6e-h). Radicicol treatment did not decrease protein levels of N-Gsdma3 and Gsdma3^Rim3^ in transfected HEK293T cells nor affecting the association of Hsp90/Hsp70 with N-Gsdma3 or Gsdma3^Rim3^ (Fig. 6i). As a positive control for Hsp90 inhibition, we found that the protein level of AKT, a well-known Hsp90 client, was decreased in a radicicol concentration-dependent manner in HEK293T cells (Fig. 6j). Hsp90 has been shown to deliver mitochondrial preproteins to the mitochondria through the interaction with Tom70 [26]. Indeed, we found that Tom70 was present in the N-Gsdma3 immunoprecipitation complex (Fig. 6k). We performed the proteinase K protection assay in the fractionated mitochondria isolated from N-Gsdma3-transfected HEK293T cells treated with vehicle or radicicol (Fig. 6l). Quantitative results revealed that the mitochondrial import of N-Gsdma3 was prevented by radicicol (Fig. 6m). Collectively, our data indicated that N-Gsdma3 utilizes the Hsp90/Hsp70 machinery for mitochondrial targeting through Tom70.

### Inhibition of ROS generation and mitochondrial permeability transition ameliorates the cell death induced by Gsdma3 mutants

**Fig. 7:**
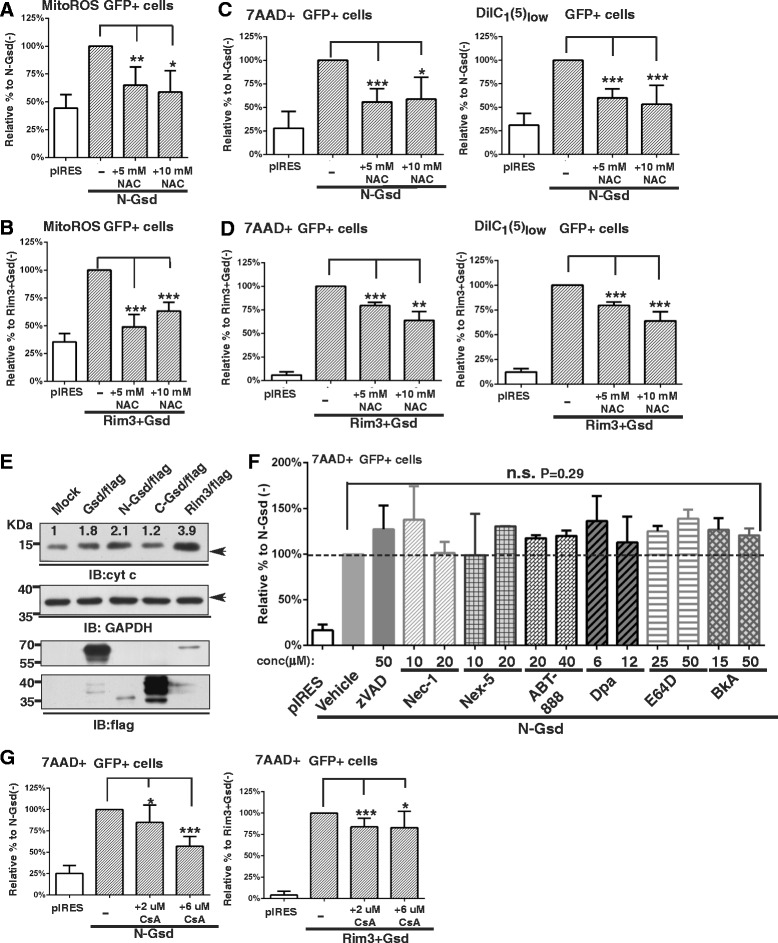
Inhibition of ROS and the mitochondrial permeability transition alleviates cell death induced by Gsdma3 mutants. (**a**, **b**) Quantification results for MitoSox Red staining. Bar graphs depict the mitochondrial ROS levels (mean + s.d., n = 3 in duplicates) in NAC-treated transfected cells relative to vehicle-treated transfected cells. (**c**, **d**) Quantification results for 7AAD and DiIC_1_(5) staining. Bar graphs depict the fractions of dead (7AAD+) cells and mitochondrial membrane potential depolarization (DiIC_1_(5)_low_) cells (mean + s.d., n = 3 in duplicates) in NAC-treated transfected cells relative to vehicle-treated transfected cells. (**e**) Immunoblot analysis of cytochrome c release in the cytosolic fraction of transfected HEK293T cells. GAPDH was used as a loading control. Relative densitometry levels normalized to GAPDH expressions are indicated. (**f**) Quantification of 7-AAD staining in bar graphs representing the fraction of dead (7AAD+) cells (mean + s.d., n = 3) in inhibitor-treated N-Gsdma3 transfected cells relative to vehicle-treated N-Gsdma3 transfected cells. n.s., not significant, P = 0.29. (**g**) Quantification results of 7AAD staining. Bar graphs depict the fraction of dead (7AAD+) cells (mean + s.d., n = 5 in duplicates) in CsA-treated N-Gsdma3 transfected cells relative to vehicle-treated 2N-Gsdma3 transfected cells. pIRES vector-transfected cells were used as the staining baseline. *, P < 0.05; **, P < 0.01; ***, P < 0.001

The association of N-Gsdma3 with Trap1 led us to investigate the mechanism of cell death induced by Gsdma3 mutants. Trap1 has been shown to prevent mitochondrial oxidative stress and modulate the mitochondrial permeability transition (MPT) [27, 28]. Therefore, we first examine whether oxidative stress is involved in cell death induced by Gsdma3 mutants. We found that treating the N-Gsdma3- and Gsdma3^Rim3^-expressing cells with the ROS scavenger NAC significantly reduced mitochondrial ROS generation (Fig. 7a, b) as well as fractions of cell death and cells with a low △ΨM (Fig. 7c, d), although the suppressive effect of NAC on Gsdma3^Rim3^-expressing cells was not as great as that observed in N-Gsdma3-expressing cells. Our results suggest that N-Gsdma3 alters the cytoprotective ability of Trap1 against oxidative stress and that ROS generation precedes △ΨM dissipation.

Excessive mitochondrial oxidative stress has been involved in the cytochrome c release from the mitochondria and MPT pore opening [29, 30]. Indeed, we observed elevated level of cytochrome c release into the cytosol in Gsdma3-, N-Gsdma3-, and Rim3-trasnfected HEK293T cells (Fig. 7e). We therefore investigated whether caspase-dependent apoptosis or other cell death mechanism is involved in the cell death induced by Gsdma3 mutants (Fig. 7f). Using pharmacological inhibitors for key mediators in apoptosis and necroptosis pathways [31], we first found that the pan-caspase inhibitor z-VAD-fmk did not block the cell death induced by N-Gsdma3. In addition, we found no evidence of inhibiting cell death when treating N-Gsdma3-expressing cells with inhibitors for RIPK1 (Necrostain-1), necrosis (Necrox-5), PARP (ABT-888), acid sphingomyelinase (desipramine), pan-calpain and cathepsins proteases (E64D), and adenine nucleotide translocase (bongkrekic acid) (Fig. 7f). Interestingly, treating N-Gsdma3- and Gsdma3^Rim3^-expressing cells with a cyclophilin D inhibitor, cyclosporin A (CsA), reduced fractions of cell death (Fig. 7g). The suppressive effect of CsA on Gsdma3^Rim3^-expressing cells was not as great as that observed in N-Gsdma3-expressing cells, suggesting that Gsdma3^Rim3^ may induce unregulated MPT [27, 32]. In summary, our data indicate that the Gsdma3 mutants can induce caspase-independent cell death by oxidative stress-mediated MPT.

## Discussion

In this study, we provide evidence that Gsdma3 contains a functional N-terminal domain (N-Gsdma3) and a regulatory C-terminal domain (C-Gsdma3). Mutations in the C-Gsdma3 disrupt the association between its N- and C-terminal domains. The unmasked N-Gsdma3 associates with Hsp90 and is delivered to mitochondria via Tom70, where it induces mitochondrial ROS production and cell death. Co-expressing C-Gsdma3 with the Gsdma3 mutants abrogated their cytotoxicity. Furthermore, pharmacological interventions of mitochondrial translocation, ROS production, and MPT pore opening alleviate the cell death induced by Gsdma3 mutants. Our data suggest a novel function of Gsdma3 in modulating mitochondrial oxidative stress, which potentially involves in cellular differentiation and cell death.

All of the Gsdma3 mutants tested in our study appeared to exhibit a lower expression level than wild-type Gsdma3, suggesting that the dominant phenotype might be caused by haploinsufficiency. However, our further analyses suggest that these dominant mutations are gain-of-function mutations. The dominant mutations in Gsdma3 mutants may expose their N-Gsdma3 and then enhance the exposure of N-Gsdma3 of wild-type Gsdma3, a dominant-active effect. Importantly, our findings readily explain why the *Dfl-* and *M1Btlr*-type mutations of *Gsdma3*, which generate C-terminal truncation mutants, induce dominant phenotypes similar to these missense mutations in animals [9, 18]. Intriguingly, we noted that different amounts of C-Gsdma3 were required to alleviate the cytotoxicity of Gsdma3 mutants. As suggested by Takana *et al* [15], different locations of the mutation sites in Gsdma3 may generate differential impact on the active conformation of Gsdma3, accounting for the slightly different onset of phenotypic defect.

To investigate the mode of action of Gsdma3, we examined the subcellular localization of N-Gsdma3 and found that N-Gsdma3 exhibited a perinuclear punctate pattern that partially colocalized with fragmented mitochondria. Mitochondrial fission is known to segregate damaged or dysfunctional mitochondria for degradation [33], consistent with our finding that N-Gsdma3 induced larger fractions of cells with △ΨM depolarization. Because Gsdma3^Rim3^-expressing cells displayed more cell death than N-Gsdma3-expressing cells, the reduced colocalization of Gsdma3^Rim3^ mutant protein with mitochondria may be due to the random distribution of the mutant protein prior to cell death, a secondary effect resulting from stronger toxicity of Gsdma3^Rim3^. We noted that Gsdma3 does not contain an apparent N-terminal mitochondrial targeting sequence that could be recognized by Tom20 for mitochondrial import. Instead, our data suggest that N-Gsdma3 is delivered to the mitochondria by the Hsp90/Hsp70/Hop complex via the mitochondrial importer Tom70, which recognizes the internal targeting sequences for mitochondrial import [34]. Hsp90/Hsp70 has been shown to deliver a subset of mitochondrial preproteins to Tom70 [26], and the formation of preprotein import intermediate after docking with Tom70 can be prevented by the Hsp90 inhibitor [35], consistent with the results of our proteinase K protection assay. Pharmacological intervention of Hsp90 may also decrease Ripk1 protein level, which accounts for the inhibiting effect of radicicol on cell death induced by Gsdma3 mutants. However, inhibiting Ripk1 directly by necrostatin-1 did not rescue cell death induced by N-Gsdma3, indicating that mitochondrial targeting is crucial for Gsdma3 activation.

Mitochondrial ROS, mainly superoxide anions, are thought to be produced by electrons leaking from the mitochondrial respiratory chain. Thus, increased mitochondrial ROS production likely reflects impaired oxidative phosphorylation and is accompanied by △ΨM depolarization [36]. Our finding that N-Gsdma3 associates with the mitochondrial chaperone Trap1 may explain the underlying mechanism of mitochondrial dysfunction caused by Gsdma3 mutants. Trap1 has been shown to associate with components of the electron transport chain [37–39] as well as with CypD [40], a crucial regulator of the MPT. Moreover, silencing *Trap1* with siRNA increases ROS production, while Trap1 overexpression attenuates cell death induced by oxidative stress [28, 41]. Thus, we hypothesize that N-Gsdma3 alters Trap1 chaperone activity, which causes mitochondrial oxidative stress, △ΨM dissipation, and MPT pore opening, ultimately leading to mitochondrial failure and cell death. In the extreme situation, obliterating Trap1 activity by Gsdma3^Rim3^ might result in the formation of unregulated MPT pore by aggregated/misfolded proteins [27, 32], which cannot be reversed completely by the ROS scavenger NAC or the CypD inhibitor CsA.

Overexpression of GSDMA and Gsdma3 has been reported to induce caspase activity in gastric cell lines and to upregulate caspase 3 expression in keratinocyte cell lines, respectively [6, 16]. Interestingly, our data indicate that Gsdma3 mutant can also induce caspase-independent MPT-mediated cell death. Accumulating evidence has shown that the extent of MPT determines the fate of the stressed cells [42]. If MPT happens to a moderate degree, the cell may undergo apoptosis, whereas if MPT happens to a larger degree, the cell may undergo necrotic cell death due to bioenergetic crisis. It is likely that overexpression of Gsdma3 (a regulatable form) induces transient MPT pore opening, which causes the cytochrome c release and caspase activation, whereas overexpression of Gsdma3 mutant (a constitutively activated form) induces sustained MPT opening, which causes non-selective release of proapoptotic and pronecrotic factors as well as uncoupling of oxidative phosphorylation. Because the protein expression level in the physiological state can only partially be compared with that in a transient transfection cell culture system, the enormous cell death observed *in vitro* is less likely to occur in cells expressing Gsdma3 or its mutant *in vivo*.

Previous studies showed that *Gsdma3* is expressed in the differentiating keratinocytes of the epidermis [9, 15]. Basal keratinocytes leave the basement membrane and start to differentiate, during which they withdraw from the cell cycle and become dead corneocytes by a specialized cell death program referred to as cornification [43]. Mitochondrial ROS has been shown to play a role in epidermal differentiation [44] and cyctochrome c release during keratinocyte differentiation was suggested to activate gene expression [45]. Therefore, imbalance of the differentiation and cell death processes can compromise the skin barrier formation and cause skin disease. We surmise that cancer cells often reside in hypoxia and inflammatory microenvironment, which causes redox imbalance and possibly can induce the conformation alteration of GSDMA3. In contrast, GSDMA3 is kept in a closed conformation in normal epithelial cells, and it may sensitize epithelial cells in response to environmental factors, such as allergens, infectious agents, or physical stress. It will be interesting in future experiments to indentify the physiological signals for exposing the N-terminal domain of Gsdma3.

## Conclusions

Our results revealed that Gsdma3 is regulated by intramolecular fold-back inhibition. The genetic mutations in the C-terminal domain of Gsdma3 are gain-of-function mutations which unmask the N-terminal functional domain of Gsdma3. Gsdma3 regulates mitochondrial oxidative stress through mitochondrial targeting.

## Additional files

Additional file 1: Figure S1. The association between Gsdma3^wt^ and Gsdma3^Rim3^ and the expression of untagged C-Gsdma3 can recue the expression of N-Gsdma3, related to Fig. 3. **(A)** Immunoblot analysis of HEK293T cells transfected with equal amounts of Gsdma3/V5 or Rim3/V5 plasmids. β-actin was used as a loading control. **(B, C, D)** Co-immunoprecipitation of Gsdma3^wt^ and Gsdma3^Rim3^. WCLs from HEK293T cells transfected with the indicated plasmid constructs were immunoprecipitated (IP) and immunoblotted (IB) with the indicated antibodies. The immunoblots were stripped and re-probed to detect the immunoprecipitated tagged constructs; WCLs (input) were immunoblotted to detect the expression of the tagged constructs. **(E)** The N-Gsdma3 protein level is upregulated by expressing C-Gsdma3 but not C-Rim3. Immunoblot analysis of HEK293T cells transfected with the indicated plasmid constructs. The molecular weight of the protein standards in kilodaltons (kDa) is indicated. Non-specific bands corresponding to the IgG heavy chain (IgGh) are indicated. Additional file 2: Figure S2. N-Gsdma3 partially colocalizes with mitochondria in 293 T kideney epithelial cells and A549 lung epithelial cells. Both 293 T cells (A) and A549 cells (B) were transfected with Gsdma3/V5 and N-Gsdma3/mh plasmids, and double immunostained for the tag and the mitochondrial marker Tom20. The color coding indicates the secondary antibody labeling. DAPI counterstains the nuclei in blue. Arrows indicate partial colocalization; arrowheads point to mitochondria; Scale bar: 20 μM. Additional file 3: Figure S3. Trap1 fragment identified with Mascot software, related to Fig. 6. Additional file 4: Table S1. Identification of proteins from 1D gel bands through nanoLC-MS/MS analysis.

## Abbreviations

Hsp: Heat-shock protein
Tom: Translocase of outer mitochondrial membrane
GSDMA: GASDERMIN A
Gsdma3: Gasdermin A3
Trap1: TNF receptor-associated protein 1
Stip1: Stress-induced-phosphoprotein 1
ROS: Reactive oxygen species
VDAC: Voltage-dependent anion channel
ANT: Adenine nucleotide translocase
CypD: Cyclophilin D
MPT: Mitochondrial permeability transition
ENU: N-ethyl-N-nitrosourea
TUNEL: Terminal dUTP nick end-labeling
IRES: Internal ribosome entry site
hrGFP: Humanized recombinant GFP
