# Supplementary figures and images for: N-terminal functional domain of Gasdermin A3 regulates mitochondrial homeostasis via mitochondrial targeting
Source: J Biomed Sci. 2015 Jun 24;22(1):44. doi: 10.1186/s12929-015-0152-0 (PMC4477613; doi:10.1186/s12929-015-0152-0)

Fig. S1

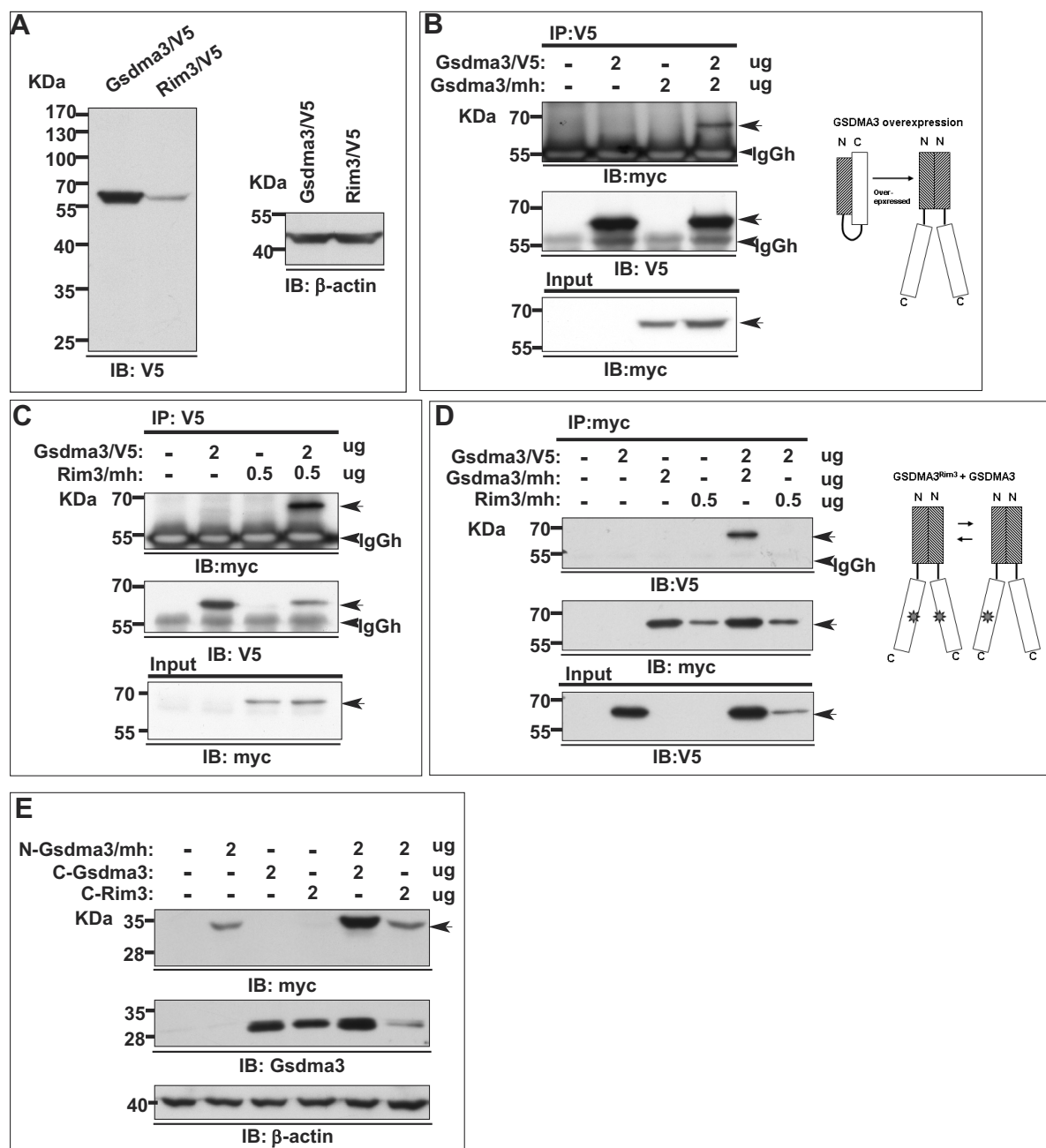

Supplement: Additional file 1: Figure S1. — The association between Gsdma3wt and Gsdma3Rim3 and the expression of untagged C-Gsdma3 can recue the expression of N-Gsdma3, related to Fig. 3. (A) Immunoblot analysis of HEK293T cells transfected with equal amounts of Gsdma3/V5 or Rim3/V5 plasmids. β-actin was used as a loading control. (B, C, D) Co-immunoprecipitation of Gsdma3wt and Gsdma3Rim3. WCLs from HEK293T cells transfected with the indicated plasmid constructs were immunoprecipitated (IP) and immunoblotted (IB) with the indicated antibodies. The immunoblots were stripped and re-probed to detect the immunoprecipitated tagged constructs; WCLs (input) were immunoblotted to detect the expression of the tagged constructs. (E) The N-Gsdma3 protein level is upregulated by expressing C-Gsdma3 but not C-Rim3. Immunoblot analysis of HEK293T cells transfected with the indicated plasmid constructs. The molecular weight of the protein standards in kilodaltons (kDa) is indicated. Non-specific bands corresponding to the IgG heavy chain (IgGh) are indicated. [file 12929_2015_152_MOESM1_ESM.pdf]

**Fig. S2****A 293T cells**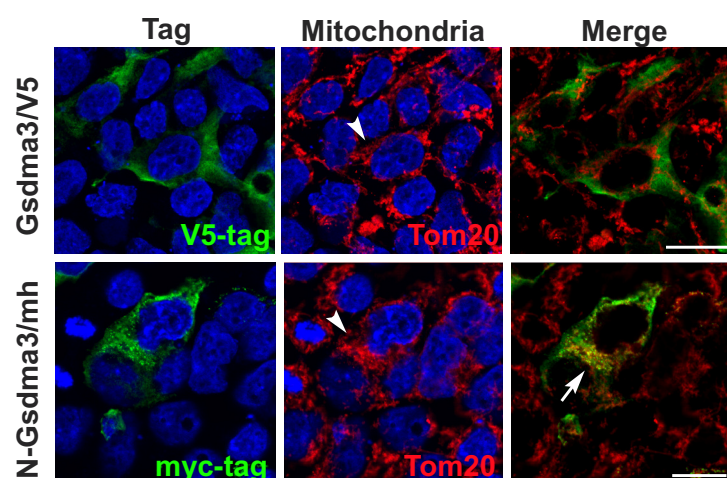**A A549 cells**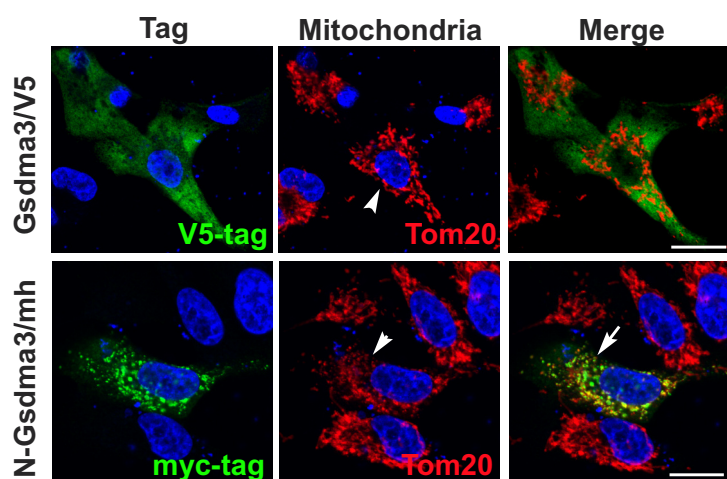

Supplement: Additional file 2: Figure S2. — N-Gsdma3 partially colocalizes with mitochondria in 293 T kideney epithelial cells and A549 lung epithelial cells. Both 293 T cells (A) and A549 cells (B) were transfected with Gsdma3/V5 and N-Gsdma3/mh plasmids, and double immunostained for the tag and the mitochondrial marker Tom20. The color coding indicates the secondary antibody labeling. DAPI counterstains the nuclei in blue. Arrows indicate partial colocalization; arrowheads point to mitochondria; Scale bar: 20 μM. [file 12929_2015_152_MOESM2_ESM.pdf]
