# Supplementary material for: N-terminal functional domain of Gasdermin A3 regulates mitochondrial homeostasis via mitochondrial targeting
Source: J Biomed Sci. 2015 Jun 24;22(1):44. doi: 10.1186/s12929-015-0152-0 (PMC4477613; doi:10.1186/s12929-015-0152-0)
Supplement: Additional file 3: Figure S3. — Trap1 fragment identified with Mascot software, related to Fig. 6. [file 12929_2015_152_MOESM3_ESM.pdf]

Table S1. Identification of proteins from 1D gel bands by nanoLC-MS/MS analysis

Gel band	Protein accession	Protein description	MW	Mascot score	Matched peptides	Coverage (%)	Peptide sequence	Ion score	m/z (observed)	Mr (calc)	ppm	Unique
1	HS90B	Heat shock protein 90-beta	83212	1153	43	50	KVVVITKH	13	329.7321	657.4425	10.8	U
							KVILHLKE	8	361.7506	721.4850	2.24	
							RLSELLRY	25	365.7292	729.4385	7.39	
							KSLVSVTKE	56	367.2279	732.4382	4.22	
							KFENLCKL (C)	36	405.6953	809.3742	2.33	
							RALLFIPRR	42	415.2688	828.5221	1.09	U
							KFYEAFSKN	33	446.2169	890.4174	2.07	U
							KTKPIWTRN	49	451.2663	900.5181	0.09	
							RYESLTDPSKL	69	520.2520	1038.4869	242	
							RAPFDLFENKK	34	540.7750	1079.5287	6.21	U
							KLGIHEDSTNRR	60	381.1935	1140.5523	5.55	U
							KYIDQEELNKT	63	576.2847	1150.5506	3.71	
							KSIYYITGESKE	26	580.7961	1159.5761	1.35	
							KIDIIPNPQERT	59	597.8283	1193.6404	1.37	U
							RRAPFDLFENKK	53	618.8226	1235.6299	0.64	U
							KADLINNLGTIAKS	64	621.8566	1241.6979	0.58	
							KEQVANSAFVERV	69	625.3147	1248.6098	4.00	U
							RELISNASDALDKI	32	638.3295	1274.6354	7.12	U
							KLGIHEDSTNRRR	70	433.2256	1296.6534	1.18	U

							KEDQTEYLEERR	48	656.2895	1310.5626	1.40	
							KHFSVEGQLEFRA	69	674.8381	1347.6572	3.34	
							RTLTLVDTGIGMTKA (M)	60	683.3695	1364.7221	1.69	U
							KEGLELPEDEEEKK	33	708.8239	1415.6303	2.05	
							RGVVDSEDLPLNISRE	96	757.4002	1512.7784	4.98	
							KSLTNDWEDHLAVKH	66	509.9202	1526.7365	1.48	
							RYESLTDPSKLDSGKE	22	513.9191	1538.7464	-7.10	
							KEGLELPEDEEEKKK	35	515.5823	1543.7253	-0.15	
							RELISNASDALDKIRY	107	515.6152	1543.8205	2.09	U
							KHLEINPDHPIVETLRQ	87	594.9921	1781.9424	6.87	U
							KHSQFIGYPITLYLEKE	44	603.6594	1807.9509	3.04	U
							RNPDDITQEEYGEFYKS	117	924.4033	924.4033	1.26	U
							KKHLEINPDHPIVETLRQ	58	637.6938	1910.0374	11.6	U
							KCLELFSELAEDKENYKK (C)	37	663.3162	1986.9244	1.19	U
							KVILHLKEDQTEYLEERR	81	672.3552	2014.0371	3.32	
							RYHTSQSGDEMTSLSEYVSRM (M)	64	731.6532	2191.9328	2.28	U
							KHNDDEQYAWESSAGGSFTVRA	61	752.6594	2254.9516	2.21	
							KLGLGIDEDEVAAEEPNAAVPDEIP PLEGDEDASRM	**70**	**1178.2205**	**3531.6376**	**0.58**	**U**
	HS90A	Heat shock protein 90-alpha	84607	981	36	40.7	KVILHLKE	8	361.7506	721.4850	2.24	U
							K.LSELLR.Y	25	365.7292	729.4385	7.39	
							KFENLCKI (C)	36	405.6953	809.3742	2.33	

							RALLFVPRR	55	408.2614	814.5065	2.15	U
							KTKPIWTRN	49	451.2663	900.5181	-0.09	
							RYESLTDPSKL	69	520.2520	1038.4869	2.42	U
							RAPFDLFENRK	44	554.7804	1107.5349	102.	U
							KYIDQEELNKT	63	576.2847	1150.5506	3.71	U
							KLGIHEDSQNRK	59	584.7899	1167.5632	1.73	U
							KELHINLIPNKQ	12	595.8441	1189.6819	-6.91	U
							RDNSTMGYMAAKK (M)	7	610.7445	1219.4849	-8.58	
							KHIYYITGETKD	18	612.8172	1223.6186	1.00	U
							KDQVANSAFVEL	74	618.3073	1234.5942	4.73	U
							KADLINNLGTIAKS	64	621.8566	1241.6979	0.58	
							RRAPFDLFENRK	94	632.8264	1263.6360	1.78	
							RELISNSSDALDKI	59	646.3237	1290.6303	1.98	U
							KEDQTEYLEERR	48	656.2895	1310.5626	1.40	
							KHFSVEGQLEFRA	69	674.8381	1347.6572	3.34	
							RTLTIVDTGIGMTKA (M)	60	683.3695	1364.7221	1.69	
							KEGLELPEDEEEKK	33	708.8239	1415.6303	2.05	
							RGVVDSEDLPLNISRE	96	757.4002	1512.7784	4.94	
							KSLTNDWEDHLAVKH	66	509.9202	1526.7365	1.48	
							RYESLTDPSKLDSGKE	22	513.9191	1538.7464	-7.10	
							KEGLELPEDEEEKKK	35	515.5823	1543.7253	-0.15	
							RYYTSASGDEMVSLKD	46	775.8592	1549.6970	4.42	

	TRAP1	Heat shock protein 75 kDa, mitochondrial	80060	96	3	6.5	RGVVDSEDIPLNLSRE	96	757.4002	1512.7784	4.94	U
2	STIP1	Stress-induced-phosphoprotein 1	62599	461	26	37.2	KDFDTALKH	30	405.2021	808.3967	8.77	U
							RIGNSYFKE	30	414.7160	827.4177	0.35	U
							RELIEQLRN	44	450.7636	899.5076	5.61	U
							KELGNDAYKK	38	455.2195	908.4239	0.56	U
							KDAIHFYNKS	30	504.2519	1006.4872	2.02	U
							KALDLDSSCKE (C)	45	504.7365	1007.4594	-0.90	U
							RLILEQMQKD (M)	42	509.7839	1017.5528	0.40	U
							RTLLSDPTYRE	53	533.2843	1064.5502	3.60	U
							KLLEFQLALKD	8	537.8308	1073.6485	-1.31	U
							KLMDVGLIAIR	71	550.8292	1099.6423	1.38	U
							RKAAALEFLNRF	61	566.8284	1131.6400	1.99	U
							KDPQALSEHLKN	39	569.2936	1136.5826	-8.73	U
							KTVDLKPDWGKG	9	579.8110	1157.6081	-0.54	U
							RIGNSYFKEEKY	45	607.8077	1213.5979	2.45	U
							KYKDAIHFYNKS	16	433.5552	1297.6455	-1.33	U
							KLDPHNHVLYSNRS	64	732.8723	1463.7269	2.12	U
							RLAYINPDLALEEKN	70	744.9035	1487.7871	3.59	U
							RTYEEGLKHEANNPQLKE	68	624.3167	1869.9220	3.33	U
							KDPQALSEHLKNPVIAQKI	77	630.0145	1887.0214	0.16	U
							KETKPEPMEEDLPENKKQ (M)	16	643.9716	1928.9037	-5.55	U

							KALSVGNIDDALQCYSEAIKL	92	1034.0072	2065.9990	0.41	U
	GRP75	Stress-70	73635	231	7	13	KVLENAEGART	16	479.7552	957.4879	8.72	U
		protein,					RRYDDPEVQKD	47	575.2816	1148.5462	2.14	U
		mitochondrial					KVQQTVQDLFGRA	60	645.8449	1289.6728	1.88	U
							RAQFEGIVTDLIRR	50	681.3749	1360.7351	0.14	U
							KLLGQFTLIGIPPAPRG	72	796.9776	1591.9450	-2.73	U
							RETGVDLTKDNMALQRV	3	569.6072	1705.8305	-18.0	U
							KSQVFSTAADGQTQVEIKV	114	904.9564	1807.8952	1.68	U
	HSP71	Heat shock 70	70009	207	6	10.6	KITITNDKG	15	402.7266	803.4389	-0.28	
		kDa protein					KDAGVIAGLNVLRI	80	599.3504	1196.6877	-1.23	U
		1A/1B					KVEIIANDQGNRT	71	614.8236	1227.6207	9.70	
							RLVNHFVEEFKRK	64	473.2590	1416.7514	2.68	U
							RTTPSYVAFTDTERL	47	744.3499	1486.6940	-5.89	
							KHWPFQVINDGDKPKV	59	560.9616	1679.8420	12.5	U
