# Supplementary material for: N-terminal functional domain of Gasdermin A3 regulates mitochondrial homeostasis via mitochondrial targeting
Source: J Biomed Sci. 2015 Jun 24;22(1):44. doi: 10.1186/s12929-015-0152-0 (PMC4477613; doi:10.1186/s12929-015-0152-0)
Supplement: Additional file 4: Table S1. — Identification of proteins from 1D gel bands through nanoLC-MS/MS analysis. [file 12929_2015_152_MOESM4_ESM.pdf]

Fig. S3

MATRIX SCIENCE Mascot Search Results

Peptide View

MS/MS Fragmentation of **GVVDSEDIPLNLSR**
Found in **TRAP1_HUMAN** in **Sprot**, Heat shock protein 75 kDa, mitochondrial OS=Homo sapiens GN=TRAP1 PE=1 SV=3

Match to Query 931: 1512.778248 from(757.396400,2+) intensity(7372574.5000) index(217)
Data file C:\Users\Administrator\Desktop\cck\L.pkl

Click mouse within plot area to zoom in by factor of two about that point
Or, Plot from 0 to 2400 Da Full range
Label all possible matches ○ Label matches used for scoring ◉
Show Y-axis ☐

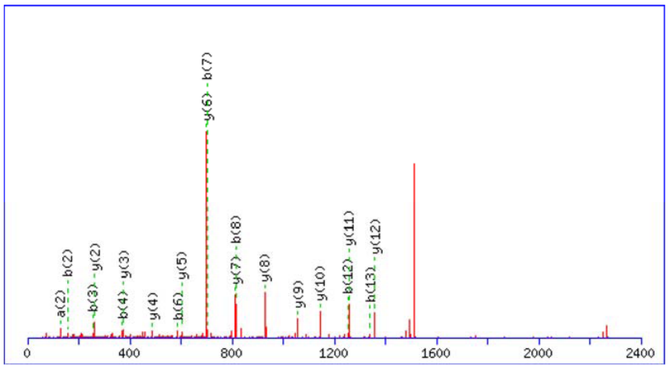

Monoisotopic mass of neutral peptide Mr(calc): 1512.7784
Ions Score: 93 **Expect:** 7.7e-009
Matches : 20/116 fragment ions using 30 most intense peaks (help)

#	a	a++	a*	a*++	b	b++	b*	b*++	Seq.	y	y++	y*	y*++	#
1	30.0338	15.5206			58.0287	29.5180			G					14
2	129.1022	65.0548			**157.0972**	79.0522			V	1456.7642	728.8857	1439.7376	720.3725	13
3	228.1707	114.5890			**256.1656**	128.5864			V	***1357.6958***	679.3515	1340.6692	670.8383	12
4	343.1976	172.1024			**371.1925**	186.0999			D	***1258.6274***	629.8173	1241.6008	621.3040	11
5	430.2296	215.6185			458.2245	229.6159			S	***1143.6004***	572.3039	1126.5739	563.7906	10
6	559.2722	280.1397			**587.2671**	294.1372			E	***1056.5684***	528.7878	1039.5419	520.2746	9
7	674.2992	337.6532			**702.2941**	351.6507			D	***927.5258***	464.2665	910.4993	455.7533	8
8	787.3832	394.1953			**815.3781**	408.1927			I	***812.4989***	406.7531	795.4723	398.2398	7
9	884.4360	442.7216			912.4309	456.7191			P	***699.4148***	350.2110	682.3882	341.6978	6
10	997.5201	499.2637			1025.5150	513.2611			L	***602.3620***	301.6847	585.3355	293.1714	5
11	1111.5630	556.2851	1094.5364	547.7719	1139.5579	570.2826	1122.5313	561.7693	N	***489.2780***	245.1426	472.2514	236.6293	4
12	1224.6470	612.8272	1207.6205	604.3139	**1252.6420**	626.8246	1235.6154	618.3113	L	***375.2350***	188.1212	358.2085	179.6079	3
13	1311.6791	656.3432	1294.6525	647.8299	**1339.6740**	670.3406	1322.6474	661.8274	S	***262.1510***	131.5791	245.1244	123.0659	2
14									R	175.1190	88.0631	158.0924	79.5498	1

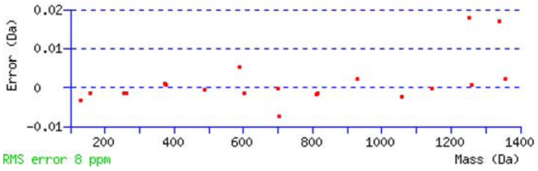

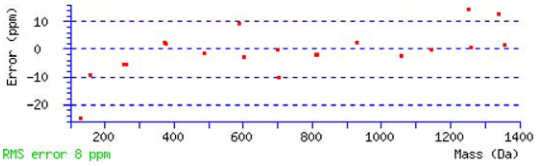

All matches to this query

Score	Mr(calc)	Delta	Sequence
93.1	1512.7784	-0.0001	GVVDSEDIPLNLSR
93.1	1512.7784	-0.0001	GVVDSEDLPLNISR
8.2	1512.8082	-0.0300	ERMGLLAVGPDLSR
5.1	1512.7507	0.0275	TIPHFCGVQLDQR
4.4	1512.7685	0.0098	KIEISGPSNFEHR
1.7	1512.7718	0.0064	ECLLAEAEGRPLR
1.6	1512.7797	-0.0014	RDVAHGILNEAYR
1.4	1512.7759	0.0024	ICLEKQPDPFAPR
1.1	1512.8024	-0.0241	KIFQIPWMHAAR
1.1	1512.7532	0.0250	VRPQEVSETEPSR
